# Efficacy of hand rubs with a low alcohol concentration listed as effective by a national hospital hygiene society in Europe

**DOI:** 10.1186/2047-2994-2-19

**Published:** 2013-06-12

**Authors:** Günter Kampf, Christiane Ostermeyer, Heinz-Peter Werner, Miranda Suchomel

**Affiliations:** 1 Bode Science Center, Bode Chemie GmbH, Melanchthonstraße 27, Hamburg, 22525, Germany; 2 Institut für Hygiene und Umweltmedizin, Ernst-Moritz-Arndt Universität Greifswald, Walther-Rathenau-Straße 49a, Greifswald, 17489, Germany; 3 Microbiology, Bode Chemie GmbH, Melanchthonstraße 27, Hamburg, 22525, Germany; 4 HygCen International GmbH, Werksgelände 24, Bischofshofen, 5500, Austria; 5 Institut für Hygiene und Angewandte Immunologie, Medizinische Universität Wien, Kinderspitalgasse 15, Vienna, 1090, Austria

**Keywords:** Hand disinfection, Efficacy, Virucidal activity, EN 1500, EN 14476

## Abstract

**Background:**

Some national hospital hygiene societies in Europe such as the French society for hospital hygiene (SFHH) have positive lists of disinfectants. Few hand disinfectants with a rather low concentration of ethanol are listed by one society as effective for hygienic hand disinfection with 3 mL in 30 s including a virucidal activity in 30 s or 60 s, but published data allow having doubts. We have therefore evaluated the efficacy of three commonly used hand disinfectants according to EN 1500 and EN 14476.

**Methods:**

Products 1 (Aniosgel 85 NPC) and 2 (Aniosrub 85 NPC) were based on 70% ethanol, product 3 (ClinoGel derma+) on 60% ethanol and 15% isopropanol (all w/w). They were tested in 3 laboratories according to EN 1500. Three mL were applied for 30 s and compared to the reference treatment of 2 × 3 mL applications of isopropanol 60% (v/v), on hands artificially contaminated with *Escherichia coli*. Each laboratory used a cross-over design against the reference alcohol with 15 or 20 volunteers. The virucidal activity of the products was evaluated (EN 14476) in one laboratory against adenovirus and poliovirus in different concentrations (80%, 90%, 97%), with different organic loads (none; clean conditions; phosphate-buffered saline) for up to 3 min.

**Results:**

Product 1 revealed a mean log_10_-reduction of 3.87 ± 0.79 (laboratory 1) and 4.38 ± 0.87 (laboratory 2) which was significantly lower compared to the reference procedure (4.62 ± 0.89 and 5.00 ± 0.87). In laboratory 3 product 1 was inferior to the reference disinfection (4.06 ± 0.86 versus 4.99 ± 0.90). Product 2 revealed similar results. Product 3 fulfilled the requirements in one laboratory but failed in the two other. None of the three products was able to reduce viral infectivity of both adenovirus and poliovirus by 4 log_10_ steps in 3 min according to EN 14476.

**Conclusions:**

Efficacy data mentioned in a positive list published by a society for hospital hygiene should still be regarded with caution if they quite obviously contradict published data on the same or similar products.

## Background

Alcohol-based hand rubs are recommended for use by healthcare workers for routine decontamination [1]. Healthcare workers usually rely on the efficacy claims provided by the manufacturer but even more on positive lists provided by infection control societies such as the VAH in Germany (Association for Applied Hygiene) [2], the ÖGHMP in Austria (Austrian Society for Hygiene, Microbiology and Preventive Medicine) [3] or the SFHH in France (French Society for Hospital Hygiene) [4]. These lists are highly appreciated by infection control practitioners because they allow an easy comparison of the efficacy of products and are considered as a quality parameter due to the neutral assessment of efficacy data. Each society has its own requirements which need to be fulfilled before a product can be listed as effective for a specific type of application. Some societies such as the SFHH require one test report per test method for the assessment, others such as the VAH and the ÖGHMP require two. The DVV (German Society for the Control of Virus Disease) organizes an additional virucidal testing in their own responsibility before issuing a certificate [5]. For hand disinfectants, the bactericidal and yeasticidal efficacy is usually determined according to the European Norms (EN) 13727, 13624 and 1500 with an application procedure resembling the use in clinical practice (e.g. 3 mL for 30 s). The virucidal activity of hand disinfectants is usually determined according to the suspension test EN 14476 with polio- and adenovirus because a test under practical conditions such as EN 1500 for bactericidal efficacy is not available yet. We have observed that some hand disinfectants with a rather low concentration of alcohol (e.g. 70% ethanol v/v) are listed as effective for hygienic hand disinfection by the SFHH which seems to contradict published data [6]. The same formulations are also listed to be virucidal in 30 s despite many published data that raise doubts [7,8]. Aim of our study was therefore to look at the bactericidal efficacy of three hand disinfectants according to EN 1500 and at their virucidal activity according to EN 14476.

## Methods

### Test products

In the positive list of the SFHH 51 products are listed as effective for hygienic hand disinfection, most of them with 3 ml for 30 s (48%), followed by 6 ml for 60 s (17%), 6 ml for 30 s (10%), 3 ml for 15 s (10%) and other applications (15%) [4]. We therefore selected from products with the most common type of application (3 ml for 30 s). The following products with a rather low concentration of alcohol were used in the study be because they are frequently used in French hospitals: Aniosgel 85 NPC, manufactured by Laboratoires Anios, Lille, France (coded as product 1), Aniosrub 85 NPC, manufactured by Laboratoires Anios, Lille, France (coded as product 2), and Clinogel derma+, manufactured by MEDA pharma, Paris, France (coded as product 3). Products 1 and 2 contain ethanol (70%, w/w), product 3 contains ethanol (60%, w/w) in combination with isopropanol (15%, w/w). All three products are listed as effective by the SFHH for hygienic hand disinfection with 3 mL in 30 s [4]. They are also described as virucidal in 30 s (products 1 and 2) or 1 min (product 3) [4].

### Bactericidal efficacy according to EN 1500

One set of experiments was performed with blinded formulations at HygCen International GmbH (Bischofshofen, Austria), one set with blinded formulations at the Institute of Hygiene and Applied Immunology of the Medical University (Vienna, Austria) and one set at Bode Chemie GmbH (Hamburg, Germany). All participants gave informed written consent. The bactericidal efficacy of each hand disinfectant was compared to the reference isopropanol 60% (v/v) in three separate cross-over experiments on artificially contaminated hands, two of them with 15 volunteers in two different laboratories (EN 1500 version 1997) [9] and one with 20 volunteers in a third laboratory (prEN 1500 version 2009) [10]. In each experiment subjects were randomly assigned to receive either test product or reference as the first application, with half of the volunteers receiving test product first, and the other half receiving the reference alcohol first. As per cross-over design, in the second application the subjects received the other product within 3 hours.

For artificial contamination, hands were washed for one min with soft soap, dried with paper towels, immersed in the *Escherichia coli* contamination fluid up to the mid-metacarpals for 5 s with fingers spread, and allowed to dry for 3 min [11]. To determine pre-decontamination values, fingertips from both hands were rubbed for one min in a separate petri dish containing liquid broth. Either 1 × 3 mL of test product or 2 × 3 mL of reference alcohol was applied to the hands. Test products were rubbed into the hands for 30 s, and reference alcohol for 2 × 30 s. The EN 1500 hand rubbing technique was used [9]. Post-decontamination values were determined immediately after the rub-in period using petri dishes containing liquid broth with neutralisers (3% Tween 80, 3% saponin, 0.1% histidine, 0.1% cysteine). For both reference and test products, log_10_ counts from the left and right hands of each subject were averaged separately, for both pre-values and post-values. The arithmetic means of all individual log_10_ reduction values were calculated. For the experiments according to EN 1500 from 1997, the Wilcoxon matched-pairs signed rank test (one-sided) was used for pair-wise comparison between mean log_10_ values obtained with test product and the reference alcohol (significance level as described in the norm, p = 0.1) [9]. For the experiments according to prEN 1500 from 2009, the Hodges & Lehmann statistics was used to evaluate for non-inferiority of the test product compared to the reference procedure. A value > 0.75 log_10_ indicates inferiority of the product to the reference procedure [10].

### Virucidal activity according to EN 14476

All experiments were performed at MikroLab GmbH, Bremen, Germany also without knowledge of the products examined. Product 1 was tested based on two blinded samples in a total of three independent test runs. Infectivity assays were done according to EN 14476 [12] with the following test viruses: poliovirus type 1 strain LSc-2ab, passaged and cultured in *BGM cells* (buffalo green monkey cells); adenovirus type 5 strain Adenoid 75, passaged and cultured in *A549 cells* (human lung epithelial carcinoma cells).

Tests were carried out in a water bath at 20°C. Three different compositions of test product, organic load and inoculum were evaluated based on the EN 14476 from 2005 [12] and the revised prEN 14476 from 2011 [13] (80 + 10 + 10; 90 + 9 + 1; 97 + 2 + 1, parts per volume). The appropriate volume of the test virus suspension and the appropriate volume of organic load (phosphate-buffered saline [PBS], Aqua bidest. for no organic load, or clean conditions [0.03% bovine serum albumin]) were mixed with the disinfectants. Clean conditions were incorporated because it was now introduced in the prEN 14476:2011 for hand rubs [13]. Immediately at the end of the chosen exposure time, activity of the disinfectant was stopped by serial dilutions with ice-cold cell culture medium. All controls required in the EN 14476 were incorporated.

Performing some determinations ready to use MicroSpin™ S-400 HR columns (GE Healthcare, Freiburg, Germany) were used in order to remove the cytotoxic agents according to instructions of the manufacturer. Examinations of the products and virus controls without columns were run in parallel.

Virus controls were incorporated after the longest exposure time. Here, the disinfectant was substituted by water of standardized hardness.

For determination of cytotoxicity of the disinfectants, the appropriate volume of Aqua bidest. was mixed with the corresponding volume of the disinfectant depending on the selected composition (final product concentration of 80%, 90%, or 97%), diluted with ice-cold cell culture medium and inoculated onto permissive cells. These controls were also performed with the different organic loads.

Infectivity was determined by means of end point dilution titration in a micro-procedure. For this, samples were diluted with ice-cold cell culture medium and 100 μL of each dilution were placed in 8 wells of a sterile polystyrene flat bottomed 96-well microtitre plate (Nunc A/S, 4000 Roskilde, Denmark) with a preformed monolayer. Cultures were observed for cytopathic effects after different days of inoculation. The infective dose (TCID_50_) was calculated according to the method of Spearman (2) and Kärber (3). Titre reduction is presented as the difference between the virus titre after defined contact time with the product and the virus titre of the control. This difference is given as log_10_ reduction. A reduction of infectivity of ≥ 4 log_10_ steps (inactivation 99.99%) was regarded as evidence for sufficient virucidal activity against the tested virus [12].

## Results

### Bactericidal efficacy according to EN 1500

Based on EN 1500 from 1997, product 1 revealed a mean log_10_ reduction of 3.87 ± 0.79 (laboratory 1) and 4.38 ± 0.87 (laboratory 2) and was significantly less effective than the reference procedure (4.62 ± 0.89 and 5.00 ± 0.87, respectively; p < 0.1; Wilcoxon matched pairs signed ranks test; Table 1). Based on prEN 1500 from 2009, the same product revealed a mean log_10_ reduction of 4.06 ± 0.86 (laboratory 3) which was inferior to the reference procedure (4.99 ± 0.90; Hodges & Lehmann value of 1.300). Product 2 revealed a mean log_10_ reduction of 3.95 ± 0.75 (laboratory 1) and 4.29 ± 0.76 (laboratory 2) and was significantly less effective than the reference procedure (4.65 ± 0.97 and 5.00 ± 0.87, respectively; p < 0.1; Wilcoxon matched pairs signed ranks test). Based on prEN 1500 from 2009, the product revealed a mean log_10_ reduction of 4.04 ± 0.92 (laboratory 3) which was inferior to the reference procedure (4.93 ± 0.46; Hodges & Lehmann value of 1.365). Product 3 revealed a mean log_10_ reduction of 3.99 ± 1.04 (laboratory 1) and 5.30 ± 0.94 (laboratory 2). In laboratory 1 it was significantly less effective than the reference procedure (4.65 ± 0.97; p < 0.1; Wilcoxon matched pairs signed ranks test), in laboratory 2 it was as effective as the reference procedure (5.15 ± 0.75). Based on prEN 1500 from 2009, the product revealed a mean log_10_ reduction of 4.27 ± 0.72 (laboratory 3) which was inferior to the reference procedure (4.99 ± 0.90; Hodges & Lehmann value of 1.095).

**Table 1:** Efficacy of three alcohol-based hand disinfectants; two experiments per product were performed according to EN 1500 (1997) to demonstrate a lack of superiority of the reference procedure using the Wilcoxon matched-pairs signed ranks test, one according to prEN 1500 (2009) to demonstrate non-inferiority of the test product using the Hodges & Lehmann (H&L) test

**Test product**	**Active ingredient(s) (concentration; all w/w)**	**Mean log**_**10**_**-reduction reference procedure (2 × 3 ml for 2 × 30 s)**	**Mean log**_**10**_**-reduction product (3 ml for 30 s)**	**p-value/Hodges & Lehmann value**
Product 1	Ethanol (70%)	4.62 ± 0.89	3.87 ± 0.79	p < 0.1
		5.00 ± 0.87	4.38 ± 0.87	p < 0.1
		4.99 ± 0.90	4.06 ± 0.86	H&L: 1.300
Product 2	Ethanol (70%)	4.65 ± 0.97	3.95 ± 0.75	p < 0.1
		5.00 ± 0.87	4.29 ± 0.76	p < 0.1
		4.93 ± 0.46	4.04 ± 0.92	H&L: 1.365
Product 3	Ethanol (60%), Isopropanol (15%)	4.65 ± 0.97	3.99 ± 1.04	p < 0.1
		5.15 ± 0.75	5.30 ± 0.94	n.a.
		4.99 ± 0.90	4.27 ± 0.72	H&L: 1.095

*n.a*., not applicable.

### Virucidal activity according to EN 14476

Product 1 revealed always a sufficient reduction of viral infectivity against adenovirus after 3 min, in some experiments already after 2 min but never at 60 s or 30 s (Table 2). Against poliovirus product 1 revealed no sufficient virucidal activity even after 3 min with a maximum log_10_ reduction of 0.87. Product 2 was found to be very effective against adenovirus within 30 s irrespective of its concentration (80% or 90%) or the chosen type of organic load. Against poliovirus, however, sufficient virucidal activity was not found after 3 min with a maximum log_10_ reduction of 3.25 (90% without organic load). Data with product 3 revealed a mixed picture with sufficient virucidal activity against adenovirus after 2 min when tested at 97% with clean conditions but insufficient virucidal activity after 2 min when tested at 80% with PBS. Against poliovirus the virucidal activity was insufficient after 3 min irrespective of the product concentration (80% or 97%) or the type of organic load (PBS or clean conditions).

**Table 2:** Activity of three hand disinfectants against adenovirus type 5 and poliovirus type 1 according to EN 14476 with various types of organic load and various product concentrations in the test

**Product**	**Active ingredient(s) (concentration; all w/w)**	**Test virus**	**Product concentration in test**	**Organic load**	**Mean log**_**10**_**-reduction of viral infectivity**			
					**30 s**	**60 s**	**120 s**	**180 s**
Product 1	Ethanol (70%)	adenovirus	80%	PBS	0.13	0.63	2.75	n.a.
			80%	PBS	0.88	1.75	3.25	≥ 4.25
			80%	PBS	1.13	2.75	≥ 4.25	≥ 5.00
			80%	clean conditions	1.63	3.25	≥ 4.63	n.a.
			90%	none	1.25	2.13	≥ 4.00	n.a.
		poliovirus	80%	PBS	0	0.13	n.a.	0.13
			80%	PBS	0.13	0.25	0	0
			80%	PBS	0.37	0.37	0.87	0.87
			80%	clean conditions	0	0.12	n.a.	0
			90%	none	0	0	n.a.	0.87
Product 2	Ethanol (70%)	adenovirus	80%	PBS	≥ 5.13	≥ 5.13	≥ 5.13	n.a.
			80%	clean conditions	≥ 5.38	≥ 5.38	≥ 5.38	n.a.
			90%	none	≥ 5.63	≥ 5.63	≥ 5.63	n.a.
		poliovirus	80%	PBS	0.13	0	n.a.	1.25
			80%	clean conditions	0	0.12	n.a.	0.75
			90%	none	0	1.75	n.a.	3.25
Product 3	Ethanol (60%), Isopropanol (15%)	adenovirus	80%	PBS	0	0.37	0.62	n.a.
			97%	clean conditions	0.62	1.50	≥ 3.50	n.a.
		poliovirus	80%	PBS	n.a.	0.38	0.50	0.38
			97%	clean conditions	n.a.	0	1.75	2.63

*n.a*., Data not available.

## Discussion

Our data indicate that key claims for three hand disinfectants approved in a positive list by a national society for hospital hygiene are highly questionable (Table 3) which raises some serious questions on the role of positive lists and the requirements set by the societies. These lists are highly appreciated by infection control practitioners because they allow an easy comparison of the efficacy of different products and are considered as a quality parameter due to the neutral assessment of efficacy data by academic infection control experts. All three hand disinfectants in our study have a rather low concentration of alcohols (70% ethanol or 60% ethanol and 15% isopropanol) but are nevertheless listed as effective for hygienic hand disinfection with 3 mL in 30 s. Our data support these doubts even more, especially since one data set per product was obtained according to the new prEN 1500 design (test for non-inferiority). It has been reported before that formulations with an alcohol-concentration up to 70% are likely to fail the EN 1500 efficacy requirement when applied as used in clinical practice with 3 mL in 30 s [6]. Even the WHO-recommended formulation with ethanol at 80% (v/v) fails the EN 1500 requirement when tested in the same way [14]. Formulations with 85% (w/w) ethanol, however, could repeatedly demonstrate sufficient bactericidal efficacy in the same design [15]. For the three products it is therefore difficult to accept that their efficacy claims, which are approved with 3 mL in 30 s for hygienic hand disinfection by the society for hospital hygiene, reflect their real efficacy, with all possible implications on patient safety.

**Table 3:** Overview on the efficacy of the three selected test products listed as effective by the SFHH for hygienic hand disinfection in comparison to our results

**Test product**	Active ingredient(s) (concentration; all w/w)*	**Efficacy according to EN 1500 (all 3 ml for 30 s)**		**Virucidal activity against poliovirus and adenovirus according to EN 14476**	
		**SFHH (at least one data set)**	**Our data (3 data sets)**	**SFHH (at least one data set)**	**Our data (multiple data sets)**
Product 1	Ethanol (70%)	Effective	Not sufficiently effective	Virucidal in 30 s (100%)	Not virucidal in 3 min (80% and 90%)
Product 2	Ethanol (70%)	Effective	Not sufficiently effective	Virucidal in 30 s (100%)	Not virucidal in 3 min (80% and 90%)
Product 3	Ethanol (60%), Isopropanol (15%)	Effective	Two data sets: not sufficiently effective; one data set: effective.	Virucidal in 1 min (100%)	Not virucidal in 3 min (80% and 97%)

*information based on label claims.

Regarding the virucidal activity (EN 14476) of the three products the results are even more conflicting. Two products are approved as virucidal in 30 s, one is approved as virucidal in 60 s [4]. Based on our data all three products were not virucidal within 3 min. Nosocomial infections are mainly caused by bacteria and yeasts and only a rather small proportion is caused by viruses [7]. Nevertheless, some non-enveloped viruses such as norovirus [16-18], adenovirus [19] or astrovirus [20] continue to cause serious infections in patients and sometimes even in healthcare workers. In order to break the chain of transmission it is essential that the hand disinfectant is truly active against non-enveloped viruses. It is known that it is very difficult to achieve comprehensive activity against non-enveloped viruses with alcohol-based formulations [7,8,21]. Based on our own and on published data [7] it seems very unlikely that the three hand disinfectants are indeed virucidal within 30 s or 60 s as claimed in the positive list. But healthcare workers will rely on an independent positive list of disinfectants which, based on our data, probably provides misleading information with all possible implications on patient safety and healthcare worker safety.

We cannot fully explain why the discrepancy between approved data and our data is so eminent for these three hand disinfectants but some possible explanation should be considered. Data according to the test methods were certainly provided by the manufacturer and were carefully analyzed by the SFHH before inclusion of a product in the positive list. In biological test systems results always vary to some extent so that one may sometimes have a favorable result and sometimes not. Therefore, in the prEN 14476:2011 the biometrical evaluation of two independent runs with the calculation of the average reduction factor and its 95% confidence interval is possible [13]. In addition, the testing laboratory has to keep in mind that the use of the Sephadex columns as proposed in the EN 14476 for detoxification requires an appropriate run in parallel without columns. By doing so, the lab can clearly notice whether parts of the test virus suspension from the test mixture are restrained in the columns which may result in false-positive results for the products. In the virus control without disinfectant this phenomenon is often not seen and then wrong conclusions are drawn.

The use of a Sephadex column may also be an explanation for favorable results in tests for virucidal activity. Formulations with a high own cytotoxicity may be tested with a Sephadex column which aims to reduce the cytotoxicity of the formulation. The Sephadex column will, however, also prolong the contact time between the formulation and the test virus for some minutes with all possible implications for the test result.

Furthermore, it is not allowed to test a hand disinfectant with the active ingredients increased by the factor 1.25 (100.0% testing). In the scope of the EN 14476 it is expressly mentioned that a disinfectant which is used in undiluted form is tested in 80% concentration and shall pass this test prior to further assessment (prEN 14476:2011 97% if 80% does not demonstrate the required reduction). In the SFHH list the three products tested in our study are all listed with 100% [4] indicating that a formulation was tested which was concentrated, e.g. 1.25 times. This is, however, not allowed according to EN 14476, and the products have been tested according to EN 14476. A formulation with e.g. 85% ethanol cannot be concentrated technically by 1.25 times. It may then only be tested as an 80% dilution which will result in the suspension in an ethanol concentration of 68%. A formulation with e.g. 70% ethanol may be concentrated technically by 1.25 times. It can also be tested as an 80% dilution which will result in the suspension in an ethanol concentration of 70%. The overall test result may be that the 70% ethanol formulation (100% testing) reveals the better virucidal efficacy compared to the 85% ethanol formulation (80% testing) which may result an efficacy assessment which can be described as misleading for clinical practice. That is why EN 14476 does not allow “ready to use products” to be concentrated for efficacy testing [13].

Furthermore, a manufacturer may collect data from many test laboratories and submit the most favorable one to include the product in the list.

The VAH and the ÖGHMP, for example, require two test reports from different laboratories which have to be independent of the manufacturer. This requirement lowers already the probability to be “wrong”. The DVV even initiates own tests to verify data submitted by a manufacturer [5]. We are also surprised that the SFHH listed product 1 (gel) and product 2 (liquid) as virucidal with the same application time of 30 s because both contain the same concentration of alcohol. Based on our data we found that the liquid reveals a stronger virucidal activity compared to the gel. Similar results have been described before with bacteria [22]. But still products 1 and 2 are listed with the same application time of 30 s as virucidal which seems to us quite unlikely to be realistic. In order to improve the validity of such lists we make a few proposals which will to our knowledge contribute to listed products and efficacy data that are more reproducible (Table 4). Some of the proposals are already state-of-the-art by VAH or ÖGHMP and may also be a good way for other societies.

**Table 4:** Six proposals to improve the validity of products claims and efficacy claims that are published in positive lists by infection control societies

**Parameter**	**Proposal**
Type of test method	The minimum requirement should be fulfilling the European norms if they exist.
Number of test reports	At least two independent test reports should be available for every type of claim.
Traceability of test product	In case of blinded formulations being tested, a test facility should store a sample that may be used by the society as a control in case of conflicting results and to ensure the identity of a formulation that is listed by name.
Restrictions of using a Sephadex column to avoid false positive efficacy data	In virucidal testing, use of a Sephadex column to reduce cytotoxicity should only be allowed when the initial virus titer is not high enough (according to EN 14476) and data according to the same method (e.g. EN 14476) are provided showing that there is no virus detectable after the recommended concentration and exposure time without using a Sephadex column. Virus controls with and without columns are not sufficient because a possible loss of virus in the column is mainly influenced by the ingredients of the test product.
Traceability of test product	A product sample should be submitted with the application for listing to allow verification of specific details mentioned in the application forms and the test reports (e.g. appearance of the product, smell, pH value, density or refraction index).
Procedure in case of conflicting results	If it is suspected that listed data are not reproducible elsewhere the society should get a product sample from the market and initiate its own efficacy test in an independent laboratory. In case of a major deviation of the results compared to submitted data, a careful analysis should be done to find the reason (identity of formulation, experimental details etc.) which may finally result in a change of the listed parameter.

The legal status of a hand disinfectant may also play a role. For a hand disinfectant which is approved as a medicinal product it is mandatory to evaluate and submit all efficacy data including EN 1500 etc. to the drug agency so that a complete overview of the efficacy can be substantiated. In such a case non-favorable data must also be presented and analyzed.

Our data were obtained in a laboratory setting and not under clinical conditions, so the test situation is a limitation of this study. In addition, the level of log_10_ reduction on hands to prevent nosocomial infections is under scientific debate. Nevertheless, a recent controlled prospective cross-over trial in intensive care units showed that introduction of a gel-based 62% ethanol product might improve compliance. The incidence of healthcare-associated infections, however, remained unchanged [23], suggesting that the concentration of ethanol in the gel may have been too low to prevent cross-transmission in clinical practice [24]. A hand rub with a better log_10_ reduction on hands, however, was shown to prevent nosocomial infections [25]. This supports our concerns about the efficacy of hand disinfectants with a low concentration of ethanol.

## Conclusions

Efficacy data mentioned in a positive list published by a society for hospital hygiene should still be regarded with caution if they quite obviously contradict published data on the same or similar products. Taking into account additional criteria from other societies and some of our proposals it should be possible ensure more validity of data in a positive list.

## Competing interests

Two authors are employed by Bode Chemie GmbH, Hamburg, Germany.

## Authors’ contributions

GK and CO made substantial contributions to conception and design, HPW and MS made a substantial contribution to acquisition, analysis and interpretation of data. GK was involved in drafting the manuscript, and all authors gave final approval of the version to be published.
